# Effect of skin color and other skin properties on the delivered light dose in phototherapy for neonatal hyperbilirubinemia

**DOI:** 10.1117/1.BIOS.2.3.032508

**Published:** 2025-08-08

**Authors:** Alida Johanna Dam-Vervloet, Rachel Foppen, Christian Victor Hulzebos, Nienke Bosschaart

**Affiliations:** a University of Twente, Faculty of Science and Technology, Biomedical Photonic Imaging Group, Enschede, The Netherlands; b Isala Hospital, Medical Physics Department, Zwolle, The Netherlands; c Beatrix Children’s Hospital, University Medical Center Groningen, Division of Neonatology, Department of Pediatrics, Groningen, The Netherlands

**Keywords:** hyperbilirubinemia, phototherapy, optical properties, skin color, Monte Carlo simulations, newborn infants

## Abstract

**Significance:**

Hyperbilirubinemia is a common and potentially harmful condition in newborn infants. Phototherapy is the primary treatment for critically high bilirubin levels. Yet, the impact of patient skin properties and the wavelength of the phototherapy light on the delivered light dose remains unclear.

**Aim:**

We investigate the impact of patient skin properties, i.e., skin color, hemoglobin concentration, bilirubin concentration, light scattering, epidermal thickness, and subdermal tissue layer type as well as phototherapy wavelength on the delivered light dose and efficacy of phototherapy using a Monte Carlo model.

**Approach:**

The model simulates light transport through the skin to predict the delivered dose and its effect on the reduction in total serum bilirubin concentration after 24 h of treatment ($\Delta {\mathrm{TSB}}_{0-24h}$).

**Results:**

Skin color, bilirubin concentration, and epidermal thickness significantly influenced the delivered light dose and predicted $\Delta {\mathrm{TSB}}_{0-24h}$. Light dose varied by a factor of 5.7 between light and dark skin, with respective predicted $\Delta {\mathrm{TSB}}_{0-24h}$ reductions of 40.8% and 25.6%. The optimal wavelength for phototherapy shifts from 460 nm for light skin to 470 nm for dark skin.

**Conclusions:**

These findings highlight the need for further research into personalized phototherapy protocols to enhance treatment efficacy, particularly for hyperbilirubinemic newborn infants with darker skin.

Statement of Discovery This study uses a Monte Carlo model to show how skin color, other skin properties, and wavelength affect phototherapy for newborn hyperbilirubinemia. Results highlight the need for personalized protocols, especially for infants with darker skin.

## 1 Introduction

Neonatal jaundice is a prevalent condition observed in the first days of life, affecting ∼80% of term infants, making it the most common medical issue in the first weeks of life. This condition arises from elevated levels of bilirubin in the body of newborn infants, i.e., neonatal hyperbilirubinemia. Although typically benign, excessively high bilirubin levels can lead to severe complications, including brain damage or death.^1^^–^^3^

The standard treatment for neonatal hyperbilirubinemia is phototherapy (PT) in case the total serum bilirubin (TSB) levels exceed the predefined treatment threshold according to the applied hyperbilirubinemia management guideline.^4^ PT facilitates the breakdown of bilirubin into more water-soluble forms that can be excreted easily from the body. As such, PT has been shown to effectively reduce TSB levels and thereby the need for exchange transfusions.^5^ The recently published American Academy of Pediatrics Hyperbilirubinemia Guideline recommends a PT irradiance of at least $30\;\mu W\;{\mathrm{cm}}^{-2}\;{\mathrm{nm}}^{-1}$ for intensive PT with blue light, which spectrally overlaps with the absorption peak of bilirubin around 450 nm.^4^^,^^6^ Beyond the gestational and postnatal age and certain hyperbilirubinemia or neurotoxicity risk factors, current PT guidelines adopt a uniform approach, independent of skin color. Although in general PT works well in newborn infants with dark skin, the uniform application hereof disregards individual variations in patient skin properties, whereas these properties are known to affect light penetration and, consequently, the delivered light dose.^7^^–^^9^

To investigate whether PT guidelines would need adaptation toward a more skin-specific approach, this study evaluates the impact of the variation in optical skin properties and PT wavelength on the delivered light dose and efficacy of PT for neonatal hyperbilirubinemia. Hereto, a Monte Carlo simulation model is employed to investigate the effects of (1) skin color, (2) skin hemoglobin concentration, (3) skin bilirubin concentration, (4) reduced scattering coefficient of the skin, (5) epidermal thickness, and (6) subdermal tissue layer type. Previous research has shown that most of these factors also influence transcutaneous bilirubin (TcB) measurements, which are performed in the same wavelength range as PT on neonatal skin.^10^^–^^13^

## 2 Methods

### 2.1 Monte Carlo Model

**Fig. 1:**
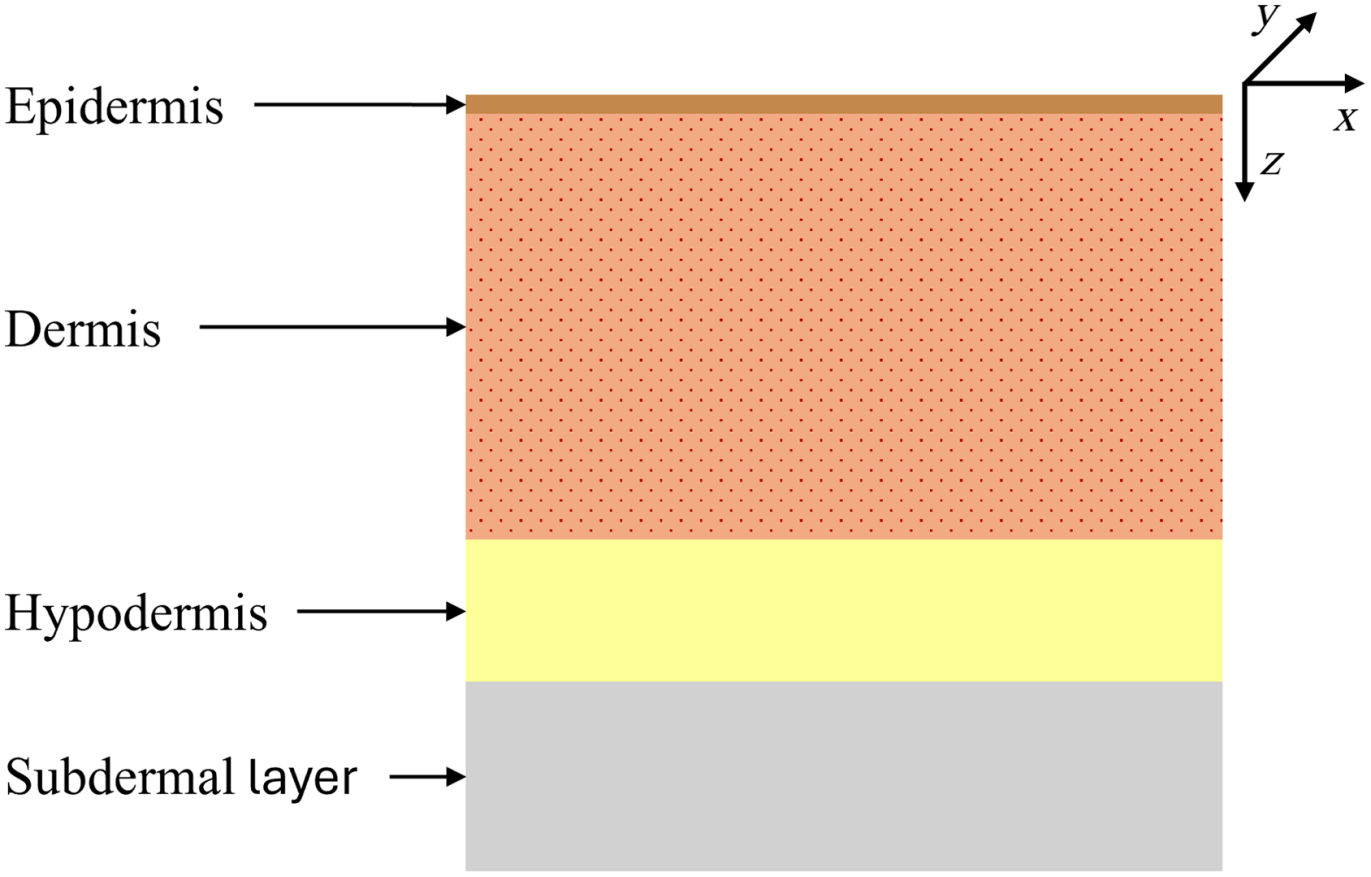
Schematic representation of the four-layer neonatal skin model.

Monte Carlo models are widely used to simulate light transport through the skin and can be used to predict the delivered light dose and efficacy of PT.^14^^–^^17^ For this study, we employed MC MATLAB,^18^ which is an open-source 3D Monte Carlo light transport solver that is based on well-established Monte Carlo codes.^14^ As input for the Monte Carlo (MC) simulation model, the skin was modeled as a semi-infinite medium consisting four different layers: the epidermis, dermis, hypodermis, and subdermal layer (Fig. 1). Standard layer properties (Table 1) were defined based on the average physiological and optical properties of neonatal skin, and the average thickness of each skin layer,^19^^–^^28^ as further detailed in Secs. 2.2.1–2.2.6. Using these standard layer properties as a simulation basis, the input parameters were varied independently within the biological range to investigate the influence of the following factors on the delivered light dose: (1) epidermal melanin absorption, (2) dermal hemoglobin absorption, (3) dermal bilirubin absorption, (4) (epi)dermal light scattering, (5) epidermal thickness, and (6) subdermal tissue layer. In addition, the simulation wavelength was varied within the therapeutic range (Sec. 2.2.7). PT efficacy was defined as the predicted reduction of TSB during 24 h of PT (Sec. 2.4).

**Table 1:** Input values for the optical properties and thickness per simulated skin layer in the Monte Carlo model. All optical properties are defined for a wavelength of 460 nm, similar to the wavelength where bilirubin light absorption is maximal. The standard layer properties represent the average neonatal skin properties (Standard model). For each simulated parameter, designated as a varied skin property series, the simulated variations in skin properties are given with respect to the standard model.

	Parameter	Epidermis	Dermis	Hypodermis	Subdermal tissue
Standard model					
Standard layer properties	$\mu_{a}$ (${\mathrm{cm}}^{-1}$)	19.6	11.9	8	15
	$\mu_{s}^{\prime }$ (${\mathrm{cm}}^{-1}$)	20.9	20.9	39.4	43.4
	g (−)	0.75	0.75	0.80	0.92
	n (−)	1.37	1.37	1.44	1.55
	d (μm)	40	900	300	360
Varied skin property series					
1. Epidermal melanin absorption	$\mu_{a}$ (${\mathrm{cm}}^{-1}$)	1.7 to 404	—	—	—
2. Dermal hemoglobin absorption	$\mu_{a}$ (${\mathrm{cm}}^{-1}$)	—	11.3 to 12.7	—	—
3. Dermal bilirubin absorption	$\mu_{a}$ (${\mathrm{cm}}^{-1}$)	—	2.5 to 14.4	—	—
4. (Epi)dermal light scattering	$\mu_{s}^{\prime }$ (${\mathrm{cm}}^{-1}$)	11.1 to 30.6	11.1 to 30.6	—	—
5. Epidermal thickness	d (μm)	20 to 180	—	—	—
6. Subdermal tissue layer type	$\mu_{a}$ (${\mathrm{cm}}^{-1}$)	—	—	—	1.2 to 15.0
	$\mu_{s}^{\prime }$ (${\mathrm{cm}}^{-1}$)	—	—	—	8.7 to 43.4
	g (−)	—	—	—	0.80 to 0.92
	n (−)	—	—	—	1.37 to 1.55

### 2.2 Optical Properties and Phototherapy Wavelength

#### 2.2.1 Epidermal melanin absorption (skin color)

In the epidermis, melanin is the primary absorber, making the absorption coefficient predominantly dependent on melanin concentration.^19^^,^^25^ In the absence of epidermal absorption values for neonatal skin, we base the absorption coefficient of the epidermis, $\mu_{a,\text{epidermis}(\lambda )}$ on the values for adults from Jacques:^19^

$$\mu_{a,\text{epidermis}(\lambda )}=f_{\text{melanosomes}}\cdot \mu_{a,\text{melanosomes}(\lambda )}+(1-f_{\text{melanosomes}})\cdot \mu_{a,\text{epidermis},\text{baseline}(\lambda ),}$$ (1)

where $f_{\text{melanosomes}}$ is the volume fraction of melanosomes, $\mu_{a,\text{melanosomes}(\lambda )}$ is the absorption coefficient of melanosomes, and $\mu_{a,\text{epidermis},\text{baseline}(\lambda )}$ is the baseline absorption coefficient of the epidermis without melanosomes. The baseline absorption is^19^

$$\mu_{a,\text{epidermis},\text{baseline}(\lambda )}=7.84\cdot 10^{8}\cdot \lambda^{-3.255},$$ (2)

and the absorption coefficient of melanosomes is^19^

$$\mu_{a,\text{melanosomes}(\lambda )}=6.6\cdot 10^{11}\cdot \lambda^{-3.33},$$ (3)

For the standard model, a melanosome volume fraction of 2% was used, corresponding to a $\mu_{a,\text{epidermis}(\lambda )}$ of $19.6\;{\mathrm{cm}}^{-1}$ for 460 nm, representing light skin.

The melanosome volume fraction in adult skin ranges from 1.3% to 6.3% for light skin, to 11% to 16% for moderately pigmented skin, and 18% to 43% for darkly pigmented skin.^19^ Using Eqs. (1)–(3), this results in a range of 1.7 to $404\;{\mathrm{cm}}^{-1}$ for $\mu_{a,\text{epidermis}(\lambda )}$ at a wavelength of 460 nm.

#### 2.2.2 Dermal hemoglobin absorption

In the dermis, red blood cells and bilirubin are the primary absorbers,^26^ where optical absorption by red blood cells is mainly caused by the chromophores Hb and ${\mathrm{HbO}}_{2}$.^23^ Therefore, the absorption coefficient of the dermis depends on the blood volume fraction in the skin, oxygen saturation ${\mathrm{SO}}_{2}$, hematocrit, and the amount of intra- and extravascular bilirubin. The absorption coefficient of the dermis, $\mu_{a,\text{dermis}(\lambda )}$, is defined as

$$\mu_{a,\text{dermis}(\lambda )}=f_{\text{blood}}\cdot \mu_{a,\text{blood},\mathrm{Hb}(\lambda )}+c_{\text{bilirubin}}\cdot \mu_{a,\text{bilirubin}(\lambda ),}$$ (4)

where $f_{\text{blood}}$ is the dimensionless volume fraction of blood in the dermis, $\mu_{a,\text{blood},\mathrm{Hb}(\lambda )}$ is the absorption coefficient of whole blood in ${\mathrm{cm}}^{-1}$ due to the presence of hemoglobin, $c_{\text{bilirubin}}$ is the combined intra- and extravascular concentration bilirubin in the dermis in μmol/L, and $\mu_{a,\text{bilirubin}(\lambda )}$ is the absorption coefficient of bilirubin in ${\mathrm{cm}}^{-1}/(\mu \mathrm{mol}/L)$. In this model, the scaling of the intravascular bilirubin concentration with $f_{\text{blood}}$ is ignored due to the negligible contribution of the intravascular bilirubin absorption to the total $\mu_{a,\text{dermis}(\lambda )}$ (∼1%).^25^ We further define

$$\mu_{a,\text{blood},\mathrm{Hb}(\lambda )}={\mathrm{SO}}_{2}\cdot \mu_{a,\mathrm{Hb},\mathrm{SO}2>98\%}(\lambda )+(1-{\mathrm{SO}}_{2})\cdot \mu_{a,\mathrm{Hb},\mathrm{SO}2=0\%}(\lambda ).$$ (5)

Literature spectra of $\mu_{a}$ for oxygenated ($\mu_{a,\mathrm{Hb},\mathrm{SO}2>98\%}(\lambda ))$ and deoxygenated ($\mu_{a,\mathrm{Hb},\mathrm{SO}2=0\%}(\lambda )$) blood with a hematocrit of 45% were used to calculate $\mu_{a,\text{blood},\mathrm{Hb}(\lambda )}$.^23^

For the standard model, the average $f_{\text{blood}}$ of 0.35% for neonatal skin was used as an input.^25^ Assuming that a newborn infant receiving PT has an ${\mathrm{SO}}_{2}$ of 95% and a hematocrit of 45%, the average $\mu_{a,\text{blood},\mathrm{Hb}(\lambda )}$ amounts to $0.78\;{\mathrm{cm}}^{-1}$ at 460 nm.

The blood volume fraction in neonatal skin ranges from 0.1% to 0.75%.^20^ Using Eqs. (4) and (5), this results in a dermal blood absorption contribution ($f_{\mathrm{blood}}\cdot \mu_{a,\text{blood},\mathrm{Hb}(\lambda )}$) that ranges from 0.2 to $1.7\;{\mathrm{cm}}^{-1}$, leading to an $\mu_{a,\text{dermis}(\lambda )}$ between 11.3 and $12.7\;{\mathrm{cm}}^{-1}$ at 460 nm.

#### 2.2.3 Dermal bilirubin absorption

For newborn infants, the combined intra- and extravascular dermal concentration of bilirubin, $c_{\text{bilirubin}}$, is on average approximately four times lower than the TSB value in blood serum.^25^^,^^29^^,^^30^ In a patient study on 60 newborn infants, this relation was defined as^25^

$$c_{\text{bilirubin}}=0.26\cdot \mathrm{TSB}+0.90.$$ (6)

For the standard model, we used a TSB value of 340  μmol/L, which equals a common TSB value for which PT is applied.^4^^,^^30^ This results in a dermal bilirubin concentration $c_{\text{bilirubin}}$ of 89.3  μmol/L and gives an absorption^6^ contribution ($c_{\mathrm{bilirubin}}\cdot \mu_{a,\text{bilirubin}(\lambda )}$) of $11.1\;{\mathrm{cm}}^{-1}$ at 460 nm.

In combination with the absorption contribution of blood, this results in an average $\mu_{a,\text{dermis}(\lambda )}$ for the standard model of $11.9\;{\mathrm{cm}}^{-1}$ at 460 nm [Eq. (4)].

TSB-based treatment thresholds for PT in newborn infants range from 50  μmol/L to 420  μmol/L.^2^^,^^4^^,^^30^ Using Eqs. (6) and (7), this results in a range of $\mu_{a,\text{dermis},\text{bilirubin}(\lambda )}$ between 1.7 and $13.7\;{\mathrm{cm}}^{-1}$, leading to a $\mu_{a,\text{dermis}(\lambda )}$ between 2.5 and $14.4\;{\mathrm{cm}}^{-1}$ at 460 nm.

#### 2.2.4 Epidermal and dermal light scattering

The average reduced scattering coefficient $\mu_{s}^{\prime }$ of $20.9\;{\mathrm{cm}}^{-1}$ for neonatal skin at 460 nm was used in the standard model for both the epidermal and dermal layer, which was obtained from a previously published study on 60 newborn infants.^20^

The total range in $\mu_{s}^{\prime }$ was defined as 11.1 to $30.6\;{\mathrm{cm}}^{-1}$ at 460 nm, based on the mean ± three standard deviations of data from the same study.^20^ Under the assumption that the anisotropy factor of 0.753 at 460 nm for adult skin also holds for neonatal skin,^27^^,^^31^ this results in a nonreduced scattering coefficient range of $\mu_{s}=45.1$ to $124.1\;{\mathrm{cm}}^{-1}$. The average refractive index, n, of the neonatal epidermis and dermis was assumed to be 1.37.^26^

#### 2.2.5 Epidermal thickness

The average epidermal thickness of newborn infants $d_{\text{epidermis}}=40\;\mu m$ was used in the standard model.^26^ The value of $d_{\text{epidermis}}$ was varied between the minimum and maximum values reported in the literature, i.e., from 20 to 180  μm.^11^^,^^32^^–^^35^

The average thicknesses of the dermis, hypodermis, and subdermal tissue layer were 900, 300, and 360  μm, respectively.^26^ These layer thicknesses were kept constant over all simulations.

#### 2.2.6 Subdermal tissue layer

The skin is supported by the hypodermis and various types of subdermal tissues, depending on its location on the body. The subdermal tissues typically involve bone, muscle, or fat. Table 2 lists the optical properties of the hypodermis and the different tissue layers that were used as an input to the simulations.

**Table 2:** Optical properties of different subdermal tissue layer types at a wavelength of 460 nm.

	$\mu_{a}$ [${\mathrm{cm}}^{-1}$]	$\mu_{s}^{\prime }$ [${\mathrm{cm}}^{-1}$]	g [−]	n [−]
**Hypodermis**				
Hypodermis^21^^,^^22^	8.0	39.4	0.80	1.44
**Subdermal tissue layer**				
Bone^24^^,^^28^^,^^36^	15.0	43.4	0.92	1.55
Muscle^22^	1.2	8.7	0.90	1.37
Fat^21^^,^^22^^,^^26^	8.0	39.4	0.80	1.44

#### 2.2.7 Phototherapy wavelength

The influence of PT wavelength was investigated by adjusting the wavelength in the MC simulations in steps of 5 nm within the therapeutic range (450 to 520 nm), taking into account the wavelength dependency of the optical properties discussed above. Hereto, we employed Eqs. (1)–(3) to account for the wavelength-dependent behavior of the epidermal absorption coefficient for a range of melanosome volume fractions (0%, 9%, 18%, 27%, 36%, 45%). We used literature absorption spectra^6^^,^^23^ of Hb, ${\mathrm{HbO}}_{2}$, and bilirubin to determine the dermal absorption coefficient for each wavelength with the help of Eqs. (4) and (5). For the wavelength-dependent epidermal and dermal scattering, the reduced scattering spectrum for newborn infant’s skin was employed, as measured by Bosschaart et al.^20^ For the epidermal and dermal anisotropy factor (g), the wavelength-dependent equation for g described by Van Gemert et al. was used.^27^ The refractive index (n) of all layers, as well as the optical properties of the hypodermis and subdermal tissue were not varied over the specified wavelength range, due to negligible influence on the delivered light dose in the dermal layer.

### 2.3 Other Input Parameters

In the standard model, the medium was modelled as a $0.5\times 0.5\times 0.160\;{\mathrm{cm}}^{3}$ cube, composed of 11×11×1000  voxels in the x, y, and z, dimensions, as defined in Fig. 1. To simulate a semi-infinite medium, cyclic side boundaries were set, in combination with escaping top and bottom boundaries. A smoothing length scale for side boundary smoothing of 5 cm was applied to meet the advised criterion of at least 10 times the cube size in the x,y-dimension.^18^^,^^37^ To simulate the illumination conditions in PT as closely as possible, a rectangular, uniform light source with a light intensity of $30\;\mu W/cm^{2}$ was modelled that covered the entire medium with half-width x,y-dimensions of $0.25\times 0.25\;{\mathrm{cm}}^{2}$ and zero angular intensity distribution.^4^ The value of $30\;\mu W/cm^{2}$ was based on the minimum intensity required for intensive PT.^4^ Per simulation, a total of $1.10^{6}\;\text{photon}$ packets were launched. A simulation wavelength of 460 nm was applied, unless stated otherwise.

### 2.4 Delivered Light Dose and Predicted TSB Decrease

The Monte Carlo simulations yield the local fluence rate,ɸ(x,y,z) in $W/{\mathrm{cm}}^{2}$, for each voxel in the simulated skin model. Subsequently, the local absorbed power per voxel, Q(x,y,z) in $W/cm^{3}$, is calculated using

$$Q(x,y,z)=ɸ(x,y,z)\cdot \mu_{a}(x,y,z),$$ (7)

where $\mu_{a}$ is the local absorption coefficient for each skin layer. The average absorbed power in the dermis layer, $Q_{\text{dermis}}$, is retrieved by summing Q(x,y,z) over the x,y,z-dimensions of the dermis layer and normalization over the number of voxels in all dimensions

$$Q_{\text{dermis}}=\frac{\sum_{x,\text{dermis}}\sum_{y,\text{dermis}}\sum_{z,\text{dermis}}Q(x,y,z)}{n_{x,\text{dermis}}\cdot n_{y,\text{dermis}}\cdot n_{z,\text{dermis}}}$$ (8)

where $n_{x,\text{dermis}}$, $n_{y,\text{dermis}}$, and $n_{z,\mathrm{dermis}}$ are the number of voxels in the dermis in the x, y, and z dimensions, respectively. As the efficacy of PT depends only on the absorbed light dose by bilirubin, $Q_{\text{bilirubin}}$ is calculated by scaling $Q_{\mathrm{dermis}}$ to the absorption contribution of bilirubin in the dermis

$$Q_{\text{bilirubin}}=Q_{\text{dermis}}\cdot \frac{\mu_{a,\text{bilirubin}}}{\mu_{a,\text{dermis}}}.$$ (9)

Finally, the difference in the absorbed light dose by bilirubin within the range of each varied skin property was calculated as the dose ratio between the maximum and minimum value of the simulated $Q_{\text{bilirubin}}$

$$\text{dose ratio}=\frac{\max(Q_{\text{bilirubin}})}{\min(Q_{\text{bilirubin}})}.$$ (10)

To relate the delivered light dose to a prediction of the decrease in TSB, the dose-response relation by Vandborg et al.^38^ was used. This relation is based on *in vivo* TSB measurements on 151 patients undergoing LED-based PT and is given by

$$\Delta {\mathrm{TSB}}_{0-24}(\%)=22.41+0.55\cdot I,$$ (11)

where $\Delta {\mathrm{TSB}}_{0-24h}$ is the TSB decrease over 24 h and I the light irradiance in $\mu \;W{\mathrm{cm}}^{-2}\;{\mathrm{nm}}^{-1}$. Vandborg’s model was adjusted with a multiplication factor ($\frac{Q_{\text{bilirubin}}}{Q_{\text{bilirubin},\text{average}}}$) to the light irradiance, to account for differences in light propagation within the skin. This adjustment allows the model to estimate the predicted TSB decrease for different skin properties by comparing the absorbed dose by bilirubin, $Q_{\text{bilirubin}}$, with average input values

$$\Delta {\mathrm{TSB}}_{0-24}(\%)=22.41+0.55\cdot I\cdot \frac{Q_{\text{bilirubin}}}{Q_{\text{bilirubin},\text{average}}}.$$ (12)

## 3 Results

Simulations on the standard neonatal skin model, as specified in Table 1, result in an absorbed power by bilirubin in the dermis of $Q_{\text{bilirubin}}=232\;\mu W/{\mathrm{cm}}^{3}$. The corresponding predicted decrease in TSB over 24 h by Vandborg’s model amounts to $\Delta {\mathrm{TSB}}_{0-24h}=38.9\%$. Simulated variations in skin properties on the standard neonatal skin model induce changes in the values of $Q_{\text{bilirubin}}$ and predicted $\Delta {\mathrm{TSB}}_{0-24h}$, as detailed below.

### 3.1 Skin Properties

#### 3.1.1 Epidermal melanin absorption (skin color)

**Fig. 2:**
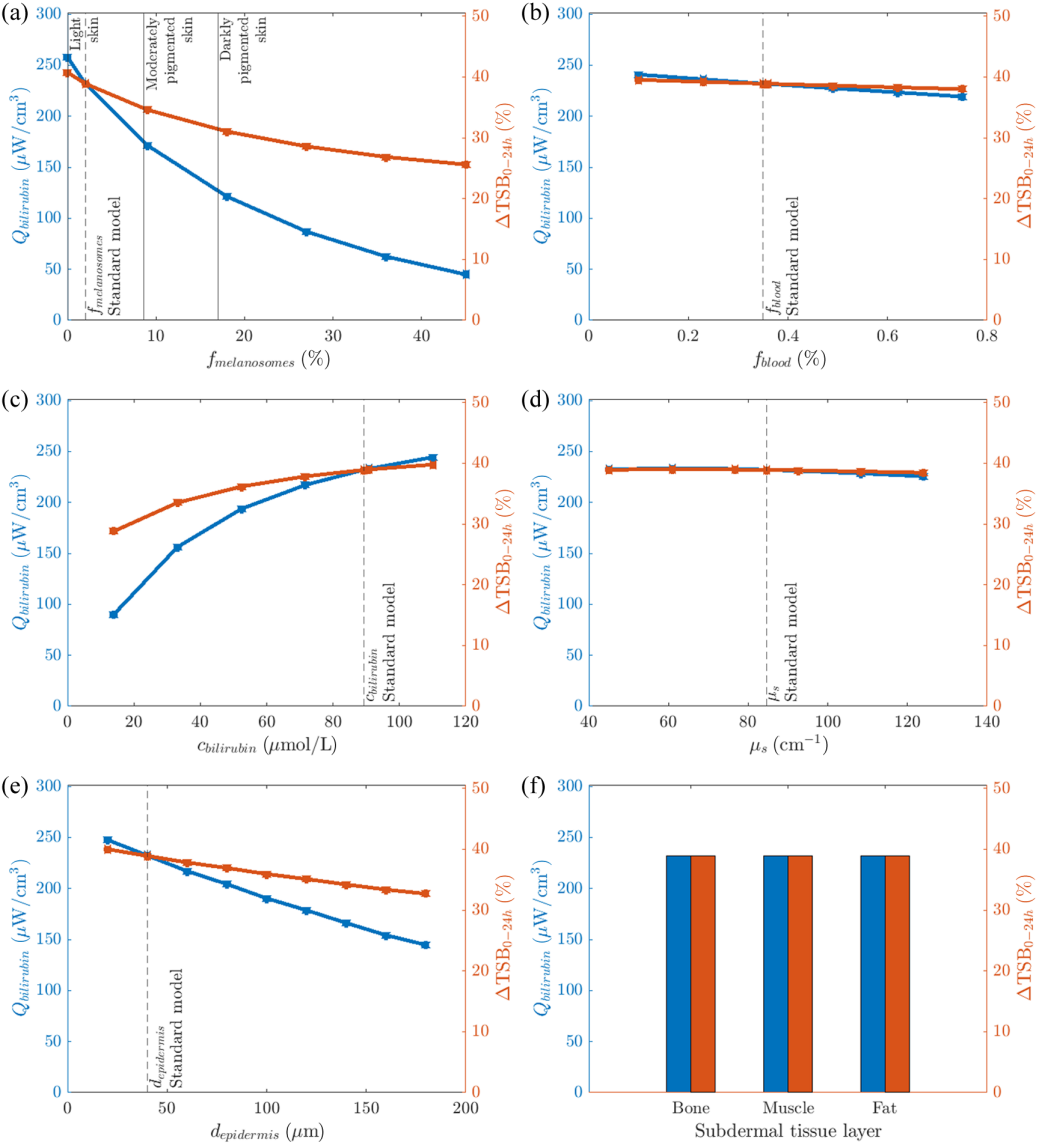
Absorbed power by bilirubin, $Q_{\text{bilirubin}}$ per ${\mathrm{cm}}^{3}$ (blue) and predicted $\Delta {\mathrm{TSB}}_{0-24h}$ (red) as a function of (a) melanosome volume fraction, $f_{\text{melanosomes}}$ in the epidermis; (b) volume fraction of blood in the dermis, $f_{\text{blood}}$ in the dermis; (c) bilirubin concentration, $c_{\text{bilirubin}}$ in the dermis; (d) light scattering $\mu_{s}$ by the (epi)dermis; (e) epidermal thickness, $d_{\text{epidermis}}$; and (f) subdermal tissue layer type.

Figure 2(a) presents $Q_{\text{bilirubin}}$ as a function of melanosome volume fraction, $f_{\text{melanosomes}}$, in the epidermis. The absorbed power by bilirubin decreases with increasing $f_{\text{melanosomes}}$ and varies between 174 and $258\;\mu W/{\mathrm{cm}}^{3}$ for light skin; 126 and $174\;\mu W/{\mathrm{cm}}^{3}$ for moderately pigmented skin; and 45 and $126\;\mu W/{\mathrm{cm}}^{3}$ for darkly pigmented skin [Fig. 2(a)]. The dose ratio between newborn infants with a light and darkly pigmented skin is 5.7. Based on Vandborg’s model, the predicted $\Delta {\mathrm{TSB}}_{0-24h}$ varies between 34.7% and 40.8% for light skin; 31.3% and 34.7% for moderately pigmented skin; and 25.6% and 31.3% for darkly pigmented skin [Fig. 2(a)].

#### 3.1.2 Hemoglobin concentration

Figure 2(b) presents $Q_{\text{bilirubin}}$ as a function of the volume fraction of blood in the dermis, $f_{\text{blood}}$. The absorbed power by bilirubin decreases with increased $f_{\text{blood}}$ and varies between 219 and $241\;\mu W/{\mathrm{cm}}^{3}$ [Fig. 2(b)]. The dose ratio between lowest and highest $f_{\text{blood}}$ is 1.1. The predicted $\Delta {\mathrm{TSB}}_{0-24h}$ varies between 38.0% and 39.5% [Fig. 2(b)].

#### 3.1.3 Bilirubin concentration

Figure 2(c) presents $Q_{\text{bilirubin}}$ as a function of the bilirubin concentration in the dermis, $c_{\text{bilirubin}}$. The absorbed power by bilirubin increases with increasing $c_{\text{bilirubin}}$ and varies between 90 and $244\;\mu W/{\mathrm{cm}}^{3}$ [Fig. 2(c)]. The dose ratio between the highest and lowest $c_{\text{bilirubin}}$ is 2.7. The predicted $\Delta {\mathrm{TSB}}_{0-24h}$ varies between 28.8% and 39.8% [Fig. 2(c)].

#### 3.1.4 Light scattering

Figure 2(d) presents $Q_{\mathrm{bilirubin}\;}$ as a function of the light scattering in the (epi)dermis, $\mu_{s}$. The absorbed power by bilirubin slightly decreases with increasing $\mu_{s}$, varying between 226 and $234\;\mu W/{\mathrm{cm}}^{3}$ [Fig. 2(d)]. The dose ratio between the lowest and highest $\mu_{s}$ is 1.04. The predicted $\Delta {\mathrm{TSB}}_{0-24h}$ varies between 38.5% and 39.0% [Fig. 2(d)].

#### 3.1.5 Epidermal thickness

Figure 2(e) presents $Q_{\text{bilirubin}}$ as a function of the epidermal thickness, $d_{\text{epidermis}}$. The absorbed power by bilirubin decreases with increased $d_{\text{epidermis}}$ and varies between 145 and $248\;\;\mu W/{\mathrm{cm}}^{3}$ [Fig. 2(e)]. The dose ratio between the lowest and highest $d_{\text{epidermis}}$ is 1.7. The predicted $\Delta {\mathrm{TSB}}_{0-24h}$ varies between 32.7% and 40.0% [Fig. 2(e)].

#### 3.1.6 Subdermal tissue layer

Figure 2(f) presents $Q_{\text{bilirubin}}$ for three different subdermal tissue layers. The absorbed power by bilirubin varies between 231.7 and $232.1\;\mu W/{\mathrm{cm}}^{3}$ among different subdermal tissue layers [Fig. 2(f)]. The dose ratio between the highest and lowest value is 1.002. The predicted $\Delta {\mathrm{TSB}}_{0-24h}$ is 38.9% for all different types of subdermal tissue layers [Fig. 2(f)].

#### 3.1.7 Phototherapy wavelength

**Fig. 3:**
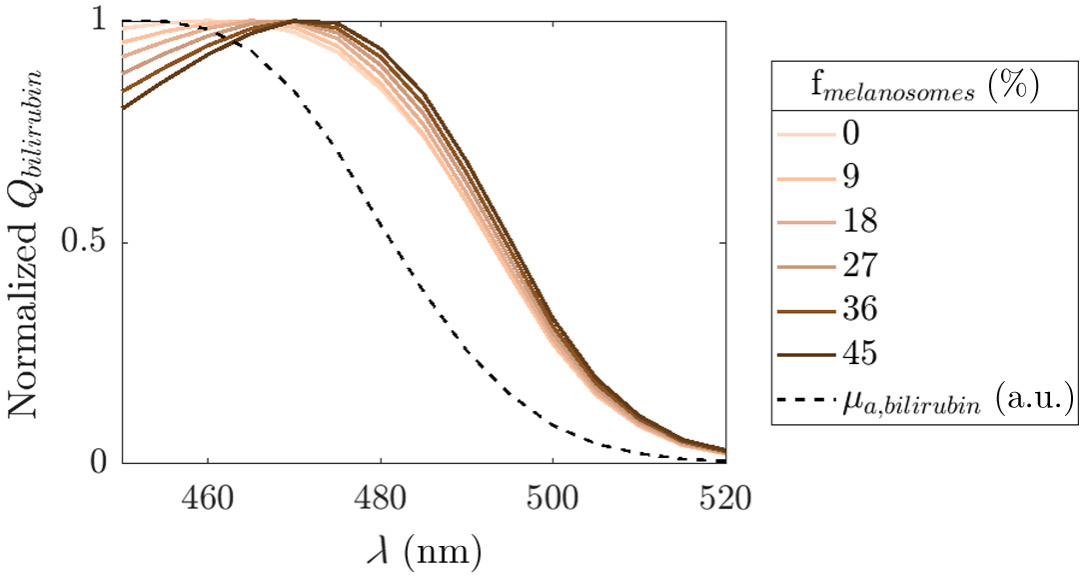
Normalized absorbed power per ${\mathrm{cm}}^{3}$ by bilirubin in the dermis as a function of wavelength, for different melanosome volume fractions ($f_{\text{melanosomes}}=0\%$; 9%; 18%; 27%; 36%, and 45%) in the epidermis.^19^ The dashed line indicates the normalized absorption spectrum of bilirubin.^6^

Figure 3 presents the normalized absorption spectrum of bilirubin and the absorbed power per ${\mathrm{cm}}^{3}$ by bilirubin in the dermis, as a function of wavelength, for different melanosome volume fractions ($f_{\text{melanosomes}}=0\%$; 9%; 18%; 27%; 36%, and 45%) in the epidermis. The wavelength at which the absorbed dose by bilirubin peaks varies from 460 nm for light skin to 470 nm for dark skin (Fig. 3).

## 4 Discussion

This study aimed to quantify the influence of patient skin (optical) properties and wavelength on the delivered light dose and predicted TSB decrease after 24 h of PT, $\Delta {\mathrm{TSB}}_{0-24}$, for neonatal hyperbilirubinemia using a well-established *in vitro* methodology with MC simulations.^39^^,^^40^ Our findings indicate that skin pigmentation has the most substantial impact on the absorbed power and predicted $\Delta {\mathrm{TSB}}_{0-24}$. An increase in skin pigmentation leads to a decrease in absorbed power by bilirubin, resulting in lower efficacy of PT for darkly pigmented newborn infants. Moreover, the MC simulations indicate that the optimal wavelength for PT is skin-specific and is higher for darkly pigmented skin types.

The main advantage of using MC simulations for this purpose is that each patient skin property can be controlled and quantified individually while keeping other skin properties constant. This is practically impossible in an *in vivo* patient setting. As the Monte Carlo model is a simplification of reality, several assumptions were made. This study assumed that bilirubin is only present in the dermis because limited information is available on bilirubin concentrations in subcutaneous tissues, in combination with minimal light penetration to these tissues. The epidermis is considered free of bilirubin due to its avascular nature.^41^

Furthermore, we utilized a model to determine the concentration of bilirubin in the skin, leveraging the relationship between TSB and TcB measurements^25^ and a dose-response model to determine the predicted $\Delta {\mathrm{TSB}}_{0-24}$.^38^ We chose these models because they are based on *in vivo* measurements on newborn infant populations of reasonable numbers. Nevertheless, it should be noted that variations between newborn infant populations may influence the physiological parameters in these models.

The skin properties hemoglobin concentration, bilirubin concentration, light scattering, and epidermal thickness were varied within the range that naturally occurs in newborn infants. It is important to note that the melanosome volume fraction of the epidermal layer was based on data from Jacques,^19^ which ranges from 0% to 43% for adult skin. Newborn infants have lower melanosome volume fractions in the epidermis compared with adults, with concentrations increasing during the first 6 months of life.^42^^–^^44^ Although there is no quantitative information available in literature on epidermal melanosome volume fractions in newborn infant skin, it is reasonable to assume that the highest concentrations modeled in this study are less common in newborn infants.

In the present work, variations in dermal thickness were neglected. As demonstrated by the negligible influence of subdermal tissue type on the absorbed light dose, the majority of light is absorbed in the epidermal and dermal layer. A greater dermal thickness will not alter these results, but this may change for very thin dermal layers. Future research exploring deeper tissue effects may therefore benefit from including dermal thickness as a variable. Furthermore, we neglected the influence of variations in tissue hydration because water absorption at 450 nm is negligible compared with the absorption by bilirubin, hemoglobin, and melanin.^45^

Similar to the outcomes of this *in vitro* study, the transmission of blue light was greatly reduced through excised, darkly pigmented epidermal layers of neonates obtained postmortem.^8^ By contrast, Porto et al.^46^ did not find a significant influence of skin color on the response of PT *in vivo*, in the first week of life. The physiological rise of TSB in the first week of life was reduced by PT in all infants, independent of ethnicity. On closer examination, however—and not discussed in Ref. 46 by the authors—the efficacy of phototherapy in non-Caucasian infants may have been lower, given that their TSB levels remained ∼1  mg/dL higher (∼20%) during treatment compared with their Caucasian counterparts. However, the study did not report data on actual skin color or hemolysis in the relatively small cohort of included newborns—both of which are essential for future research into the *in vivo* efficacy of phototherapy across varying skin tones. The simulations in the current work support our conclusion that skin color influences the delivered dose and efficacy of PT.

### 4.1 Clinical Implications

This study quantitatively analyzed factors influencing the delivered dose in PT, showing that skin properties significantly influence the light dose and predicted TSB decrease. The results of our *in vitro* study also elegantly highlight that the optimal wavelength for PT is skin-specific. Based on our results, and as a practical compromise, we speculate that a fixed wavelength near 465 nm may offer a broadly effective therapeutic window, ensuring sufficient dosing across a wide range of skin tones. A recently published randomized trial comparing two different PT wavelengths showed that blue LED light centered at 478 nm reduced bilirubin more effectively than light centered at 459 nm although effects in infants with a non-Caucasian ethnicity were not separately reported.^47^ Our results suggest that current application of similar PT intensities and spectral range in infants with different skin tones will most likely have different effects on lowering TSB levels, potentially also affecting PT duration. This study may set the stage for awareness among health care professionals to consider—next to hyperbilirubinemia risk factors—also skin-specific effects, whenever PT fails to sufficiently lower TSB levels. To determine whether a more skin-specific PT approach is needed in clinical practice, further *in vivo* studies are necessary. These studies will require quantitative assessment of the optical and anatomical skin properties of newborn infants, in combination with accurate tracking of applied PT settings (e.g., PT irradiance distribution,^9^ and PT duration) and the corresponding decrease in TSB over time for each PT session.

## 5 Conclusion

This Monte Carlo study demonstrated that patient skin properties, in particular skin color, significantly influence the delivered light dose in PT for neonatal hyperbilirubinemia. Thereby, the predicted reduction in TSB after 24 h of PT was ∼15% lower in dark skinned newborn infants compared with light-skinned newborn infants. Furthermore, the optimal wavelength for PT shifted from 460 nm for light skin to 470 nm for dark skin. These findings imply that current PT protocols may be less effective for newborn infants with darker skin colors and stress the need for more research into personalized PT protocols to optimize treatment efficacy.

## Data Availability

Open source code: https://github.com/ankrh/MCmatlab (Version 4.0.1).
